# Supplementary figures and images for: Polo-like kinase 1 regulates growth in juvenile Fasciola hepatica
Source: PLoS Pathog. 2025 Dec 2;21(12):e1013406. doi: 10.1371/journal.ppat.1013406 (PMC12688102; doi:10.1371/journal.ppat.1013406)

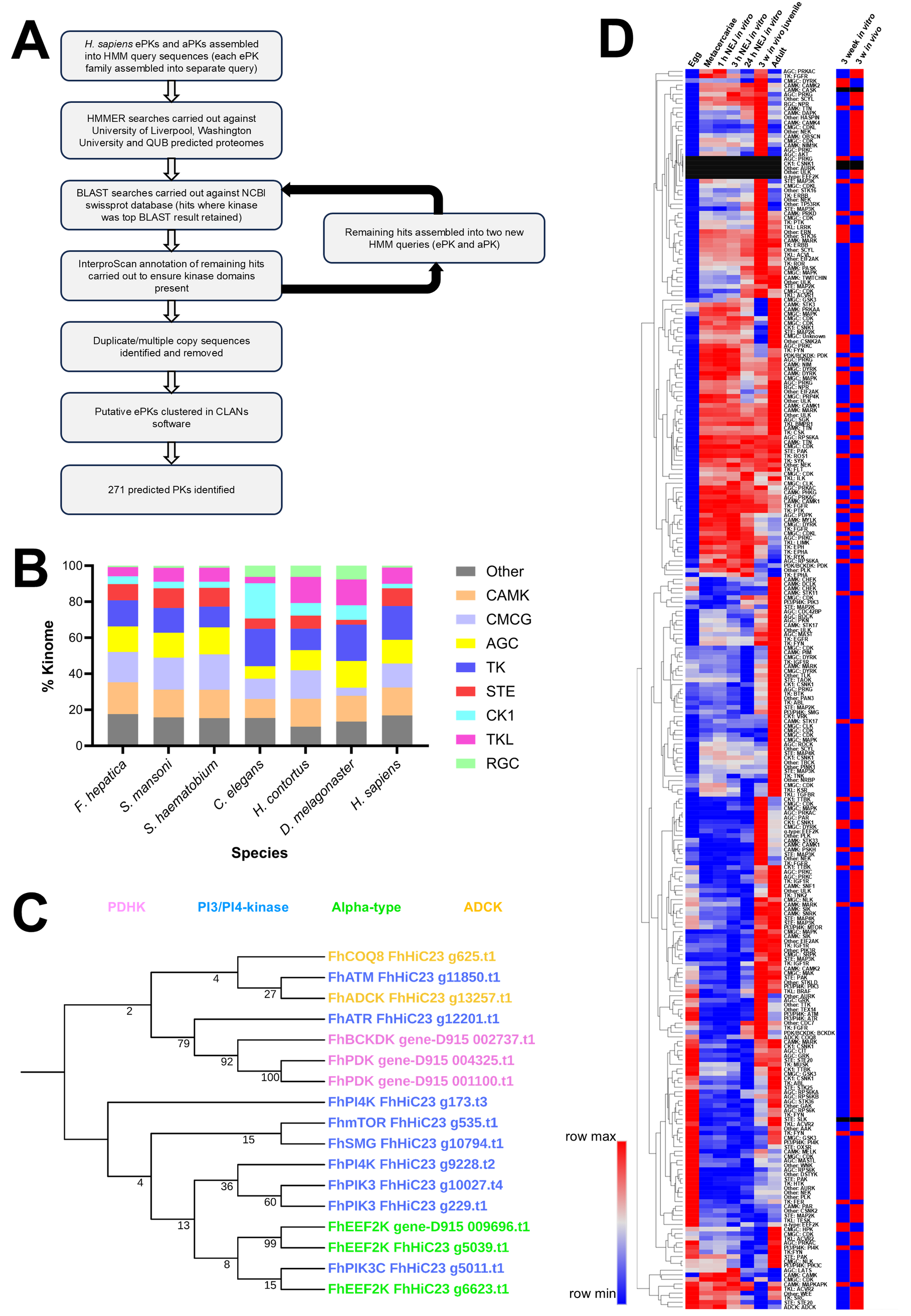

Supplement: S1 Fig — (A) Bioinformatics pipeline used to identify putative protein kinases in F. hepatica through HMM searches, BLAST identification, manual curation and CLANs analysis. (B) Proportions (%) of eukaryotic protein kinase families in F. hepatica, Schistosoma mansoni, Schistosoma haematobium, Caenorhabditis elegans, Haemonchus contortus, Drosophila melanogaster and Homo sapiens kinomes. (C) Maximum-likelihood tree (WAG + G + F model) with 100 bootstraps of predicted F. hepatica atypical protein kinases; colours denote family. (D) Heatmap of F. hepatica kinases (where data were available from WormBase ParaSite) in eggs, metacercariae, 1 h Newly excysted juveniles (NEJs) in vitro,3 h Newly excysted juveniles (NEJs) in vitro, 24 h Newly excysted juveniles (NEJs) in vitro, 3-week immature juveniles in vivo and adults (Left) with average Euclidean distancing applied shows increased expression of many kinases in NEJs and immature worms. The expression of these kinase genes was also compared between in vivo juvenile and in vitro juveniles (Right, [46]) showing increased expression of many kinases in faster growing in vivo juveniles; colours correspond to z scores (red = upregulated; blue = downregulated; grey = no change; black = no expression data). (TIF) [file ppat.1013406.s001.tif]

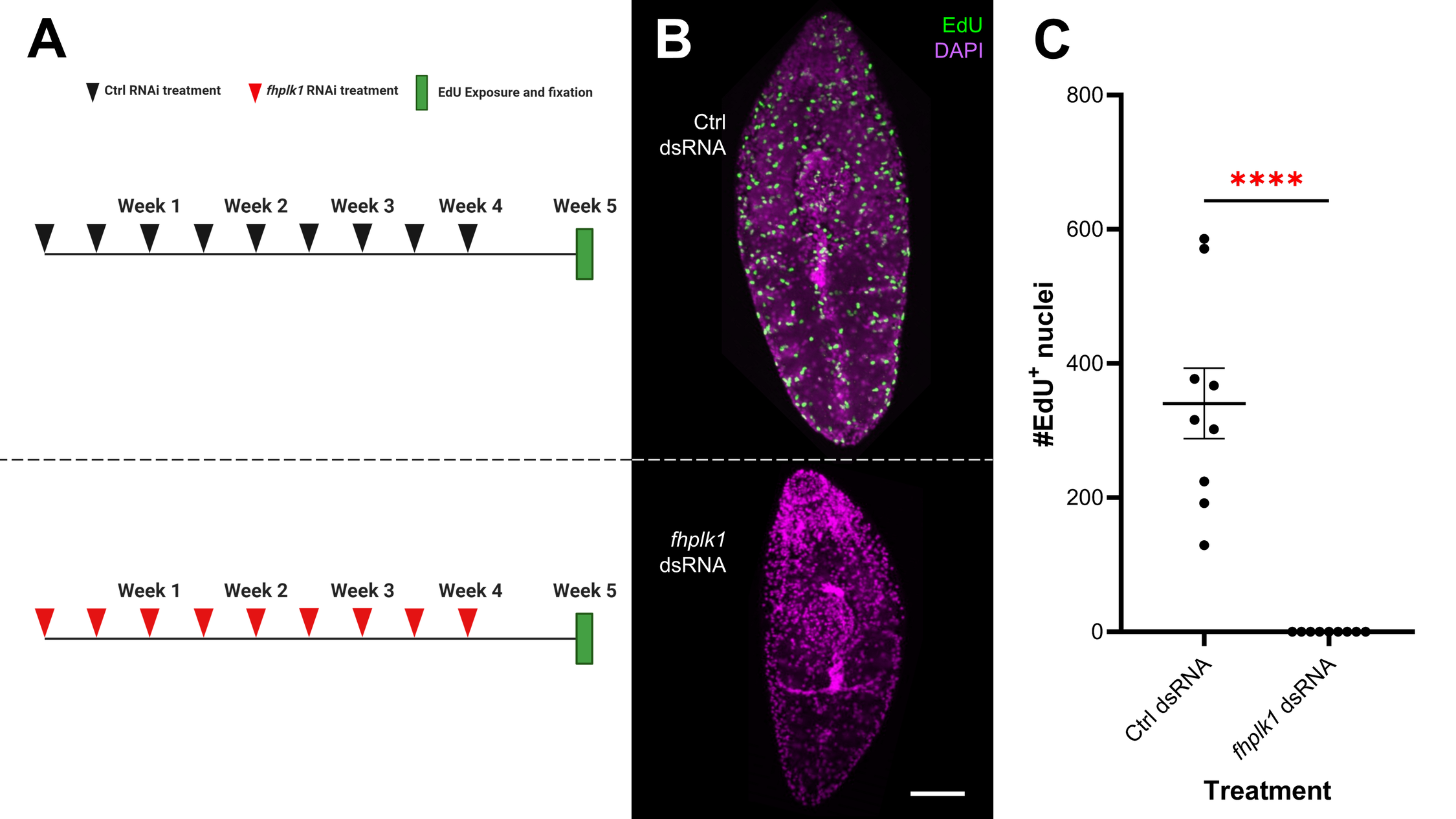

Supplement: S2 Fig — (A) Timelines for experiments show juvenile F. hepatica were treated for four weeks with either control dsRNA or fhplk1 dsRNA before a final week in culture with no dsRNA treatments prior to EdU exposure and subsequent staining. (B) Confocal images of EdU staining (green) in juvenile F. hepatica treated according to adjacent timelines show that EdU+ nuclei did not recover after fhplk1 dsRNA exposures were stopped; scale bar = 100 µm, DAPI counterstain (magenta). (C) Mean # EdU+ nuclei ±SEM in juvenile F. hepatica treated according to adjacent timelines shows complete loss of EdU+ nuclei in fhplk1 dsRNA-treated worms, even after culture for one week post drug exposure (n ≥ 8 for each treatment; Mann-Whitney U test). Experimental timelines schematic created in BioRender. (TIF) [file ppat.1013406.s002.tif]

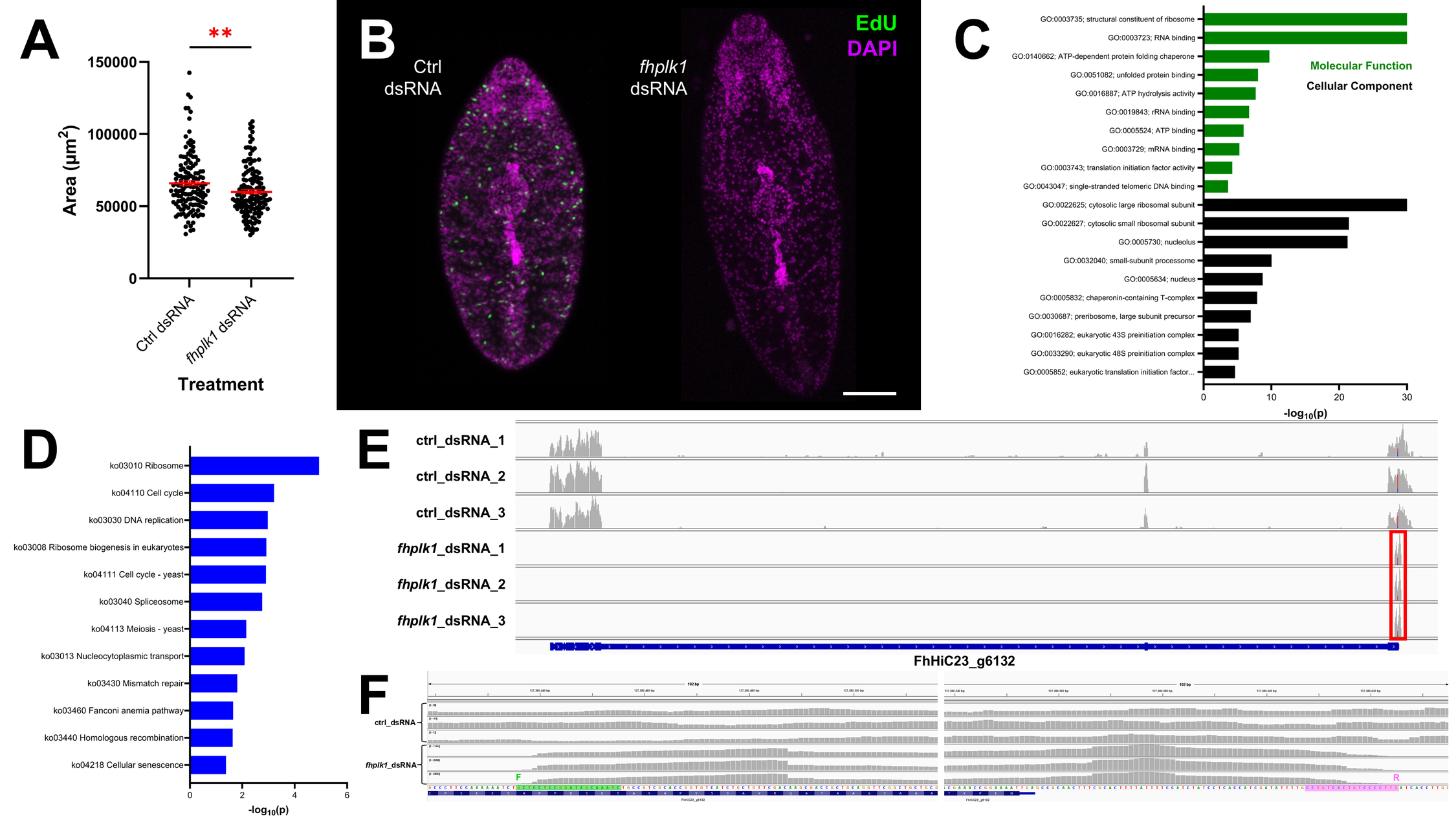

Supplement: S3 Fig — (A) Mean area (µm2) ±SEM of juvenile F. hepatica used in transcriptomics shows reduced growth rate in worms repeatedly treated with fhplk1 dsRNA for three weeks (n ≥ 141 for each treatment; Mann-Whitney U test). (B) Confocal images of EdU staining (green) in juvenile F. hepatica used for transcriptomics confirmed ablation of EdU+ nuclei after repeated treatment with fhplk1 dsRNA for three weeks. (C) GO terms (top 10 molecular function and top 10 cellular component) overrepresented among downregulated transcripts following fhplk1 dsRNA treatment in juvenile F. hepatica. (D) KEGG pathways significantly downregulated following fhplk1-RNAi in juvenile F. hepatica are associated with ribosomal components and cell cycle/proliferation. (E) Integrative genomics viewer (IGV) screenshot of transcriptomic reads in all RNA sequenced samples mapped against the F. hepatica fhplk1 gene shows reads across the whole gene in control dsRNA samples, but a spike in reads around the dsRNA amplicon region in fhplk1 dsRNA samples (red box to highlight). (F) IGV screenshots of beginning and end of fhplk1 dsRNA amplicon region show that reads in fhplk1 dsRNA samples begin and end where forward (‘F’ green) and reverse (‘R’ pink) amplicon primers are located. (TIF) [file ppat.1013406.s003.tif]

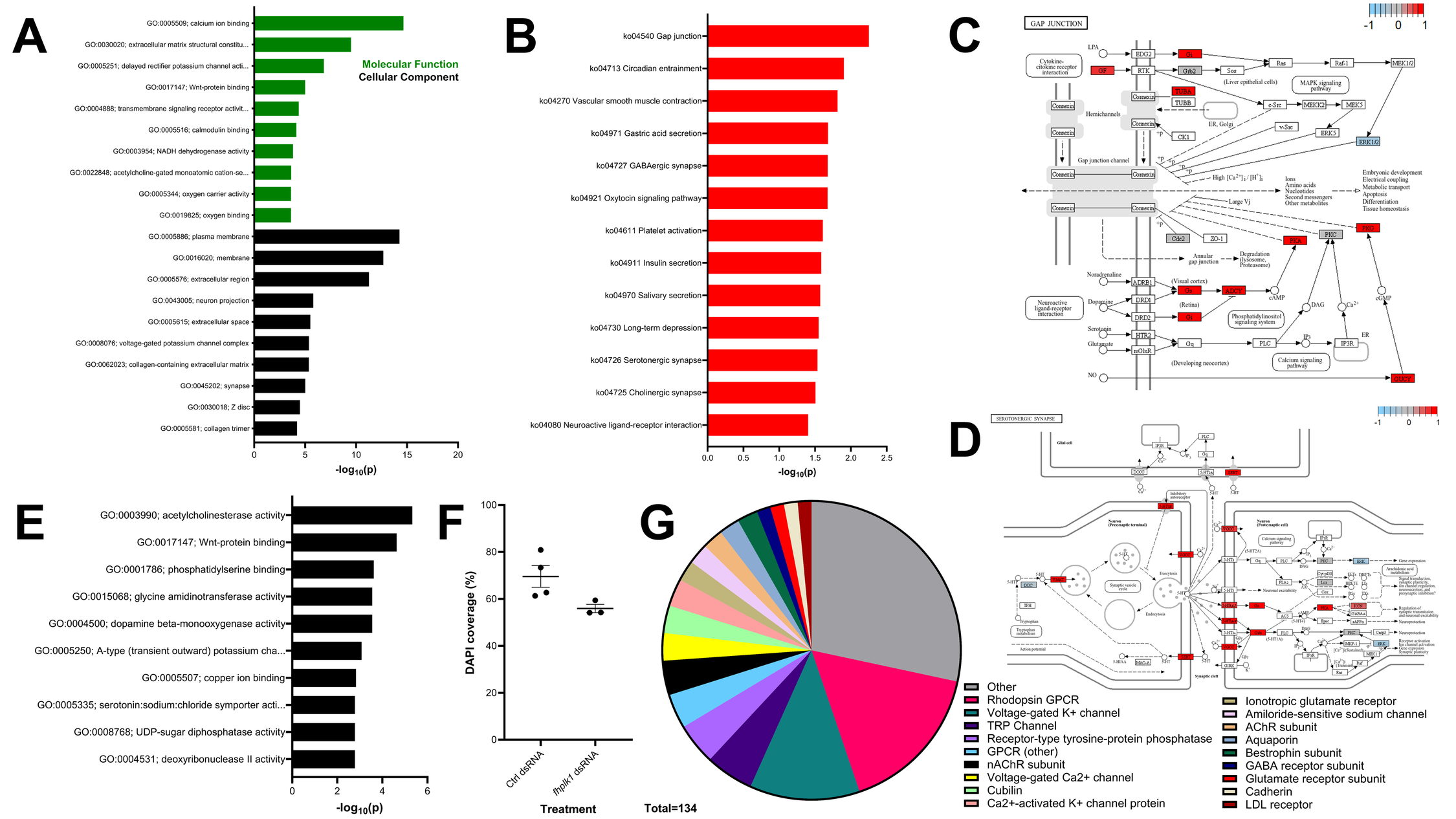

Supplement: S4 Fig — (A) GO terms (top 10 molecular function and top 10 cellular component) overrepresented in transcripts upregulated following fhplk1-RNAi in F. hepatica juveniles. (B) KEGG pathways associated with inter-cell signalling (gap junction, neural synapses and signalling pathways) significantly upregulated following fhplk1-RNAi in juvenile F. hepatica. (C) KEGG Gap Junction pathway following fhplk1-RNAi shows upregulation of diverse pathway components (red = upregulated; grey = no change; blue = downregulated; white = unassigned KEGG ID). (D) KEGG serotonergic synapse pathway following fhplk1-RNAi shows upregulation of diverse pathway components (red = upregulated; grey = no change; blue = downregulated; white = unassigned KEGG ID). (E) Top 10 Biological process GO terms overrepresented in significantly upregulated transcripts in both fhplk1-RNAi and slower growing in vitro worms (S6 Table) are associated with neuronal signalling. (F) DAPI (nuclei) stain coverage (as a percentage of the total area of the worm) in control-dsRNA treated and fhplk1-dsRNA treated worms shows ~20% reduction (n ≥ 3 for each treatment). (G) Significantly upregulated receptors/channel subunits following fhplk1-RNAi. (TIF) [file ppat.1013406.s004.tif]

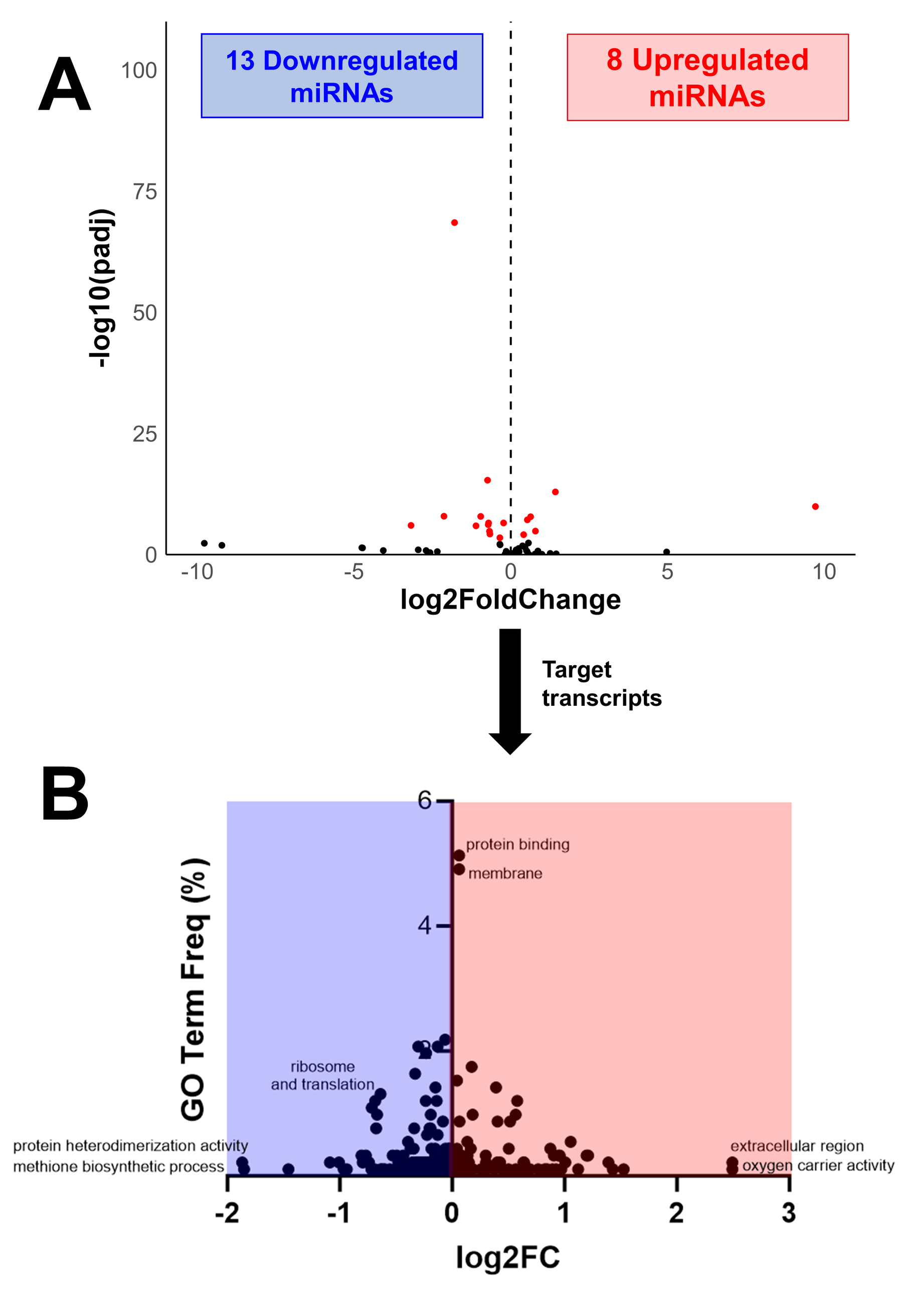

Supplement: S5 Fig — (A) Volcano plot of miRNA expression (red dots indicate differentially expressed miRNA) in fhplk1-RNAi juvenile F. hepatica. (B) Mean log2foldchange and frequency of GO terms for the predicted target transcripts of differentially expressed miRNAs following fhplk1-RNAi in F. hepatica juveniles. (TIF) [file ppat.1013406.s005.tif]

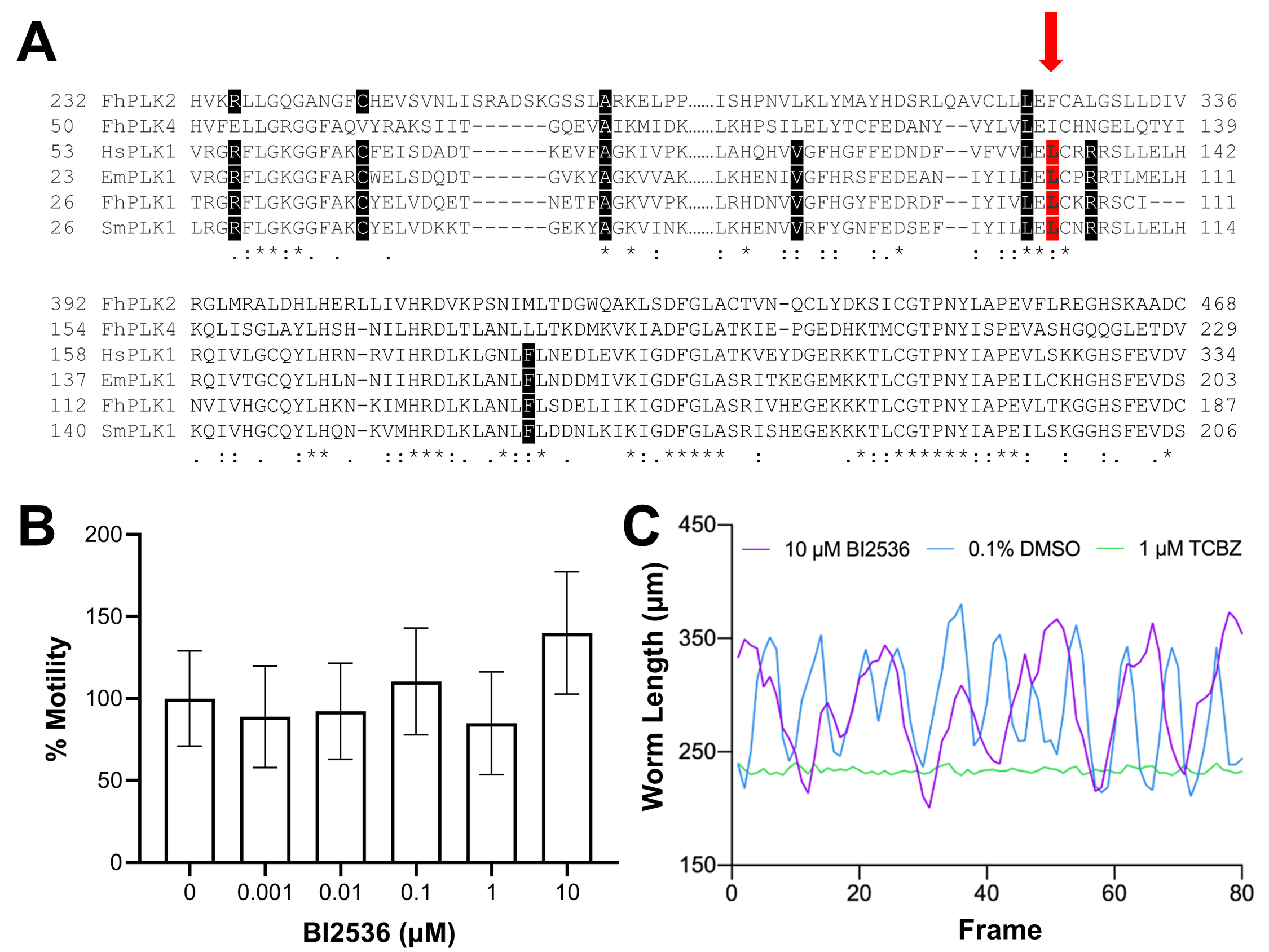

Supplement: S6 Fig — (A) Conservation of BI 2536 binding residues within F. hepatica FhPLK1, Echinococcus multilocularis EmPLK1, Schistosoma mansoni SmPLK1 and Homo sapiens PLK1 kinase domains, but not FhPLK2 or FhPLK4 (residues implicated in BI 2536 binding highlighted in black; Leu132 residue critical to drug interaction in H. sapiens PLK1 highlighted in red and marked with a red arrow). (B) Worm motility (%) ±SEM of F. hepatica newly excysted juveniles (NEJs) 18 hours after in vitro BI 2536 treatment (n ≥ 49 for each treatment). (C) Motility profile of F. hepatica NEJs treated with 10 µM BI 2536 (blue), 0.1% DMSO (purple) and 1 µM TCBZ (green). Each line represents a single parasite; data presented as worm length (µm) over time (frames). (TIF) [file ppat.1013406.s006.tif]
